# Supplementary material for: Cytoskeletal tension and Bazooka tune interface geometry to ensure fusion fidelity and sheet integrity during dorsal closure
Source: eLife. 2019 Apr 17;8:e41091. doi: 10.7554/eLife.41091 (PMC6469929; doi:10.7554/eLife.41091)
Supplement: Transparent reporting form [file elife-41091-transrepform.docx]

***eLife’s* transparent reporting form**

We encourage authors to provide detailed information *within their submission* to facilitate the interpretation and replication of experiments. Authors can upload supporting documentation to indicate the use of appropriate reporting guidelines for health-related research (see [EQUATOR Network](http://www.equator-network.org/%20)), life science research (see the [BioSharing Information Resource](https://biosharing.org/)), or the [ARRIVE guidelines](http://www.plosbiology.org/article/info:doi/10.1371/journal.pbio.1000412) for reporting work involving animal research. Where applicable, authors should refer to any relevant reporting standards documents in this form.

If you have any questions, please consult our Journal Policies and/or contact us: [editorial@elifesciences.org](mailto:editorial@elifesciences.org).

**Sample-size estimation**

- You should state whether an appropriate sample size was computed when the study was being designed
- You should state the statistical method of sample size computation and any required assumptions
- If no explicit power analysis was used, you should describe how you decided what sample (replicate) size (number) to use

Please outline where this information can be found within the submission (e.g., sections or figure legends), or explain why this information doesn’t apply to your submission:

No sample size calculation was performed. Sample size used depended on i) the extent of variability of the results and ii) the difficulty with which experiments can be repeated (most of the data presented in the paper is based on long duration live imaging and quantitative morphodynamics and some data comes from fixed preparations). Some of this information is provided in the Materials and Methods section under statistical analysis.

**Replicates**

- You should report how often each experiment was performed
- You should include a definition of biological versus technical replication
- The data obtained should be provided and sufficient information should be provided to indicate the number of independent biological and/or technical replicates
- If you encountered any outliers, you should describe how these were handled
- Criteria for exclusion/inclusion of data should be clearly stated
- High-throughput sequence data should be uploaded before submission, with a private link for reviewers provided (these are available from both GEO and ArrayExpress)

Please outline where this information can be found within the submission (e.g., sections or figure legends), or explain why this information doesn’t apply to your submission:

The detailed information is provided in the methods section for each parameter quantified, and states when a parameter was measured multiple times (to rule out human error, especially when differences are small) and also in multiple samples (stating numbers). Wherever possible, and in most graphs in the manuscript, each data point is shown. This information is provided in the methods section. All the data obtained was used in the analysis (also stated in the Methods section) with the exclusion of embryos for which imaging rendered the sample unmeasurable during the entire duration of imaging for various reasons (incomplete duration, drift etc). The figure legends also indicate how often an experiment was done.

**Statistical reporting**

- Statistical analysis methods should be described and justified
- Raw data should be presented in figures whenever informative to do so (typically when N per group is less than 10)
- For each experiment, you should identify the statistical tests used, exact values of N, definitions of center, methods of multiple test correction, and dispersion and precision measures (e.g., mean, median, SD, SEM, confidence intervals; and, for the major substantive results, a measure of effect size (e.g., Pearson's r, Cohen's d)
- Report exact p-values wherever possible alongside the summary statistics and 95% confidence intervals. These should be reported for all key questions and not only when the p-value is less than 0.05.

Please outline where this information can be found within the submission (e.g., sections or figure legends), or explain why this information doesn’t apply to your submission:

Statistical analysis methods are described in the Material and Methods section on statistical analysis. The definitions of centre (e.g. median, mean) and variation (e.g. standard deviation, interquartile range) have been mentioned both under the respective section in Materials and Methods and also in the figure legends.

Raw data (each data point) is presented in most graphs except those that indicate relative frequencies in which case the sample size from which those distributions were arrived at is stated in the figure legend.

(For large datasets, or papers with a very large number of statistical tests, you may upload a single table file with tests, Ns, etc., with reference to sections in the manuscript.)

**Group allocation**

- Indicate how samples were allocated into experimental groups (in the case of clinical studies, please specify allocation to treatment method); if randomization was used, please also state if restricted randomization was applied
- Indicate if masking was used during group allocation, data collection and/or data analysis

Please outline where this information can be found within the submission (e.g., sections or figure legends), or explain why this information doesn’t apply to your submission:

The samples are defined by their genotypes. No sample randomization was performed. No blinding was performed.

**Additional data files (“source data”)**

- We encourage you to upload relevant additional data files, such as numerical data that are represented as a graph in a figure, or as a summary table
- Where provided, these should be in the most useful format, and they can be uploaded as “Source data” files linked to a main figure or table
- Include model definition files including the full list of parameters used
- Include code used for data analysis (e.g., R, MatLab)
- Avoid stating that data files are “available upon request”

Please indicate the figures or tables for which source data files have been provided:

A description of the geometric parameters measured is provided in Fig 1 and a description of all parameters measured is provided in detail in the Materials and Methods section. Fig S2 contains additional data.
